# All‐in‐One Multispectral Stealth Device for X‐Band Radar, Visible, and Near‐Infrared Spectra

**DOI:** 10.1002/nap2.70102

**Published:** 2026-04-22

**Authors:** Jinwoo Ho, Juhyun Park, Jiyoung Oh, Chil Seong Ah, Jae Woo Seo, HeaSook Park, Tae‐Youb Kim, Sungjoon Lim

**Affiliations:** ^1^ Department of Intelligent Semiconductor Engineering Chung‐Ang University Seoul Republic of Korea; ^2^ Display and Nano System Laboratory, School of Electrical Engineering Korea University Seoul Republic of Korea; ^3^ Reality Devices Research Division Electronics and Telecommunications Research Institute Daejeon Republic of Korea; ^4^ Division of Materials Science and Engineering Hanyang University Seoul Republic of Korea; ^5^ School of Electrical and Electronics Engineering Chung‐Ang University Seoul Republic of Korea

**Keywords:** camouflage, electrochromic device, metamaterial absorber, multispectral stealth

## Abstract

Multispectral stealth is a critical technology in modern military and security applications. Conventional approaches generally address stealth in only one or two spectral bands—such as radar, visible light, or infrared (IR)—thereby limiting their effectiveness against advanced detection systems. In this study, we present, for the first time, an integrated and dynamically adaptive electrochromic system capable of achieving simultaneous stealth across the radar, near‐infrared (NIR), and visible spectra. This single‐device platform surpasses traditional independent or discrete methods in complexity, efficiency, and real‐time tunability. Our multispectral stealth device employs a vertically integrated electrochromic device (ECD) that unifies stealth functionality across all three spectral domains. Visible and NIR camouflage are realized through voltage‐controlled, real‐time modulation of reflectance, enabled by the electrochromic properties of the ECD and its embedded indium tin oxide (ITO) layers. Radar camouflage is achieved through precisely patterned ITO structures incorporated within the device, effectively suppressing electromagnetic wave reflections. To validate this approach, we performed numerical simulations and experimental characterizations using an X‐band waveguide, confirming consistent and robust multispectral stealth performance. This work represents a significant advancement in multispectral stealth technology by integrating radar, NIR, and visible light camouflage into a single, tunable platform.

## Graphical abstract

This study demonstrates an all‐in‐one multispectral stealth platform seamlessly combining an electrochromic device with a patterned ITO metasurface. The device achieves reversible, voltage‐driven visible‐to‐near‐infrared modulation through redox reactions, simultaneously maintaining X‐band radar absorption. The performance of this all‐in‐one device was verified in a waveguide, suggesting that it can be scaled up to large‐area structures.

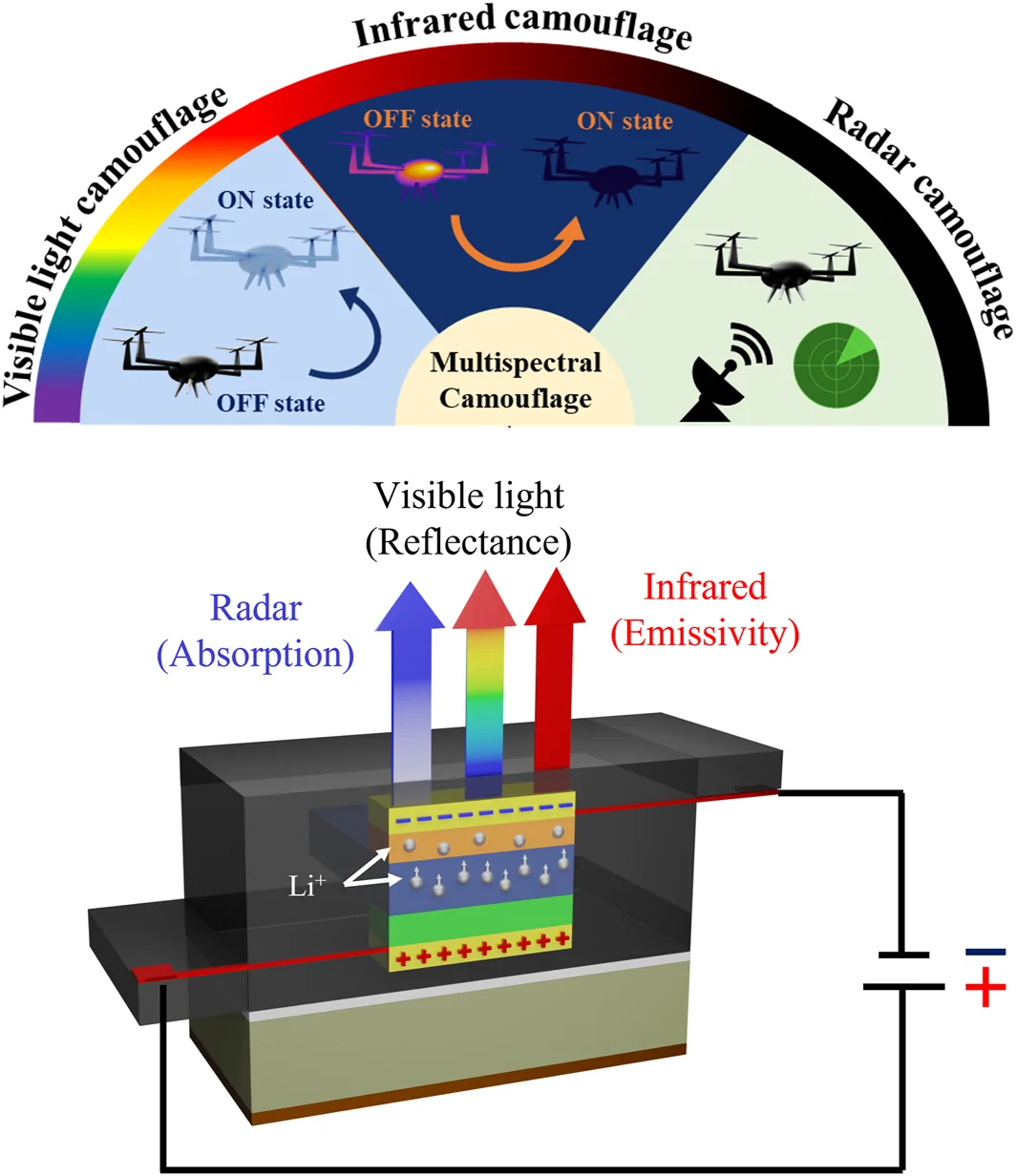

## 1 Introduction

Stealth technology has become a cornerstone of modern defense systems, crucial for enhancing the survivability of military assets against increasingly sophisticated surveillance networks [1, 2]. As detection technologies have evolved from single‐mode sensors to multispectral systems operating simultaneously across radar, infrared (IR), and visible spectra, conventional stealth methods targeting only a single band have become inadequate [3, 4]. Consequently, there is an urgent imperative to develop advanced multispectral stealth technologies capable of achieving comprehensive concealment in diverse electromagnetic environments. Although traditional approaches such as radar‐absorbing materials (RAMs) and passive camouflage paints have been widely utilized, they lack the adaptability required to counter dynamic detection threats, necessitating the transition toward intelligent, active stealth platforms [5, 6].

To address these challenges, extensive research has been conducted on developing advanced functional materials for multispectral compatibility [7, 8, 9]. Metamaterial absorbers have attracted particular attention owing to their ability to efficiently absorb electromagnetic waves within designated frequency bands [10, 11, 12, 13, 14, 15, 16]. Recent developments in artificial periodic structures have further enhanced radar stealth performance through the tailoring of electromagnetic responses [17, 18, 19, 20, 21]. Near‐Infrared (NIR) stealth is achieved by controlling surface reflectance to counter detection by night vision devices (NVDs) and active IR illumination systems. Unlike thermal stealth, which manages heat signatures via emissivity control, NIR stealth requires the object to mimic the reflection characteristics of the background environment. Visible light camouflage generally relies on active color modulation using electrochromic or thermochromic materials, which alter their optical properties to blend dynamically with the surrounding environment [22, 23]. However, these techniques typically operate independently, and integrating multiple stealth mechanisms to achieve multispectral camouflage significantly complicates device design, increases production costs, and reduces operational flexibility [24, 25]. A critical bottleneck remains in the integration of these distinct mechanisms. Existing multispectral solutions often rely on physically stacking multiple functional layers, which inevitably leads to increased device thickness, heavy weight, and impedance mismatch between layers. Furthermore, most reported devices operate passively or provide concealment in only two spectral bands such as Radar‐IR or Radar‐Visible, failing to offer a unified, real‐time adaptive solution across the three principal spectral domains [26, 27, 28, 29, 30, 31, 32, 33].

In this study, we propose an innovative, monolithic multispectral stealth device that seamlessly integrates visible, near‐infrared (NIR), and radar concealment into a single electrochromic platform. Unlike conventional multilayer stacking approaches, our design utilizes a compact all‐in‐one architecture based on a tungsten trioxide (WO_3_) and nickel oxide (NiO_x_) complementary electrochromic device (ECD). The key innovation lies in the dual functionality of the patterned Indium Tin Oxide (ITO) electrodes. They serve not only as transparent conductive agents for the ECD but also as a metamaterial absorber for X‐band radar. This strategic design allows the device to actively modulate its optical and IR properties via redox reactions whereas simultaneously absorbing radar waves through structural resonance, effectively solving the trade‐off between optical transparency and microwave absorption [7, 34].

We comprehensively evaluated the performance of the proposed device through both numerical simulations and experimental characterizations. The results demonstrate that the device achieves significant tunability in the visible and NIR regions with rapid response times, alongside efficient broadband absorption in the X‐band radar frequency. By eliminating the need for separate, bulky components, this monolithic integration simplifies the fabrication process and ensures mechanical stability. This work presents a scalable and efficient pathway for next‐generation active stealth technologies, offering a practical solution for adaptive camouflage systems in complex multi‐threat environments.

## 2 Results

### 2.1 Concept and Principle of Multispectral Stealth

Detection systems typically operate across the visible, IR, and radar (microwave) spectral bands, each characterized by distinct spectral properties and atmospheric transmittance, as illustrated in Figure 1a. Effective camouflage therefore requires simultaneous concealment across all three spectral regions.

**FIGURE 1:**
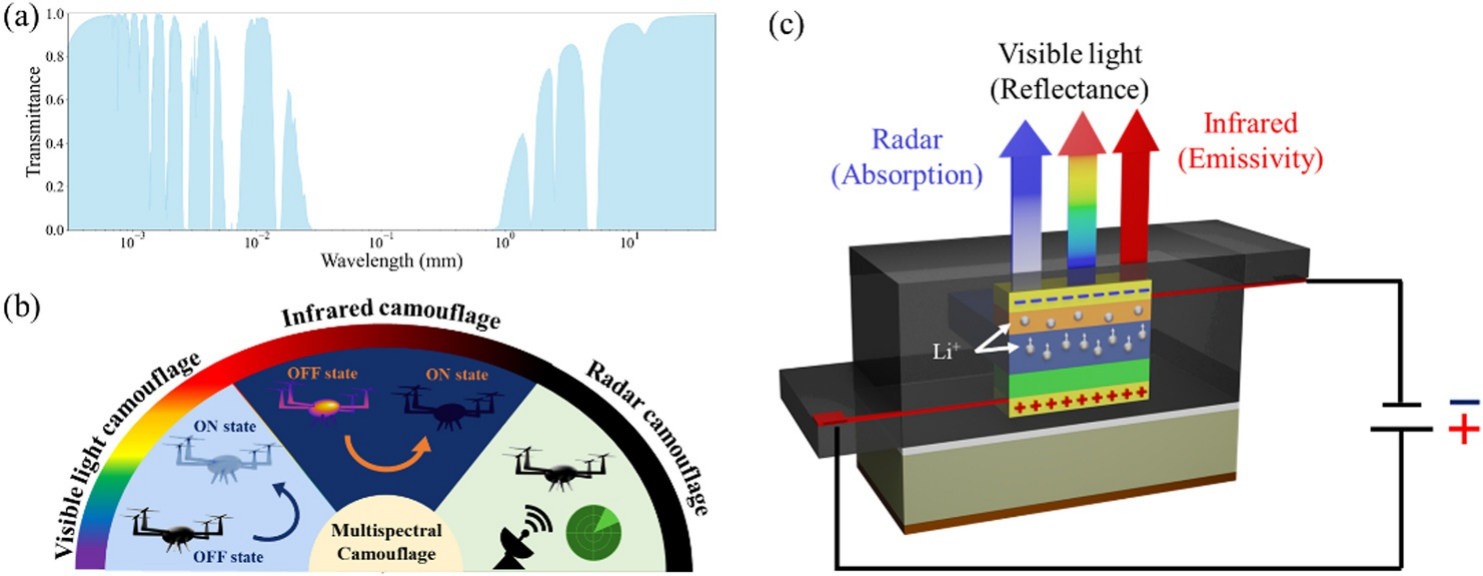
(a) Atmospheric transmittance across the microwave, IR, and visible spectral bands, alongside the corresponding operational principles of each camouflage mechanism. (b) Conceptual illustration of camouflage strategies designed for each spectral band. (c) Cross‐sectional schematic illustrating the operating mechanism of the multispectral stealth structure.

Figure 1b conceptually demonstrates the multispectral stealth capability of the proposed device when integrated onto a platform such as an unmanned aerial vehicle (UAV). Visible and NIR camouflage are achieved through the electrochromic behavior of the ECD, which enables voltage‐dependent tuning of color and thermal emissivity under a DC bias. Concurrently, radar camouflage is realized by patterning the conductive ITO layer to function as a microwave absorber.

In the visible spectrum, the ECD facilitates adaptive camouflage by dynamically adjusting its color to match the surrounding environment—for example, displaying a sky‐blue hue during aerial flight or shifting to green when flying over forested terrain. In the NIR regime, the device modulates its reflectance to match that of the background, thereby reducing its visibility to detection systems such as night‐vision goggles and NIR sensor cameras. Within the microwave region, radar stealth is achieved through the absorption of incident electromagnetic waves, effectively reducing the radar cross‐section (RCS) of the UAV.

As depicted in Figure 1c, the proposed multispectral stealth device operates on fundamentally distinct physical mechanisms in the microwave and optical regimes due to the vast disparity between the incident wavelengths and the structural dimensions. In the microwave regime (X‐band), the incident wavelength is comparable to the millimeter‐scale periodicity of the patterned ITO, enabling strong metamaterial resonances and precise impedance matching. To elucidate this, the simulated electric and magnetic field distributions clearly demonstrate the localized resonance behaviors responsible for the high X‐band absorption. In contrast, the Vis‐NIR wavelengths are several orders of magnitude smaller than the ITO features. Operating in this macroscopic limit, the structure does not support wavelength‐scale nanophotonic resonances; instead, the optical response is strictly governed by geometric optics specifically, the effective transmission through the open‐area fraction (fill factor) and the intrinsic electrochromic modulation of the active WO_3_/NiO_x_ layers. Consequently, variations in the macroscopic structural dimensions do not induce resonant spectral shifts in the Vis‐NIR range, but rather only scale the overall baseline transmittance by altering the geometric aperture ratio. Radar camouflage is achieved via a metamaterial absorption mechanism, although microwave absorption inherently converts electromagnetic energy into heat via ohmic and dielectric losses, the incident power density in practical radar detection scenarios is extremely low due to free‐space propagation loss. Even assuming an absorbed power density on the order of mW/cm^2^, the resulting steady‐state temperature rise is negligible and can be readily dissipated through substrate conduction, natural convection, and thermal radiation [35, 36, 37]. Therefore, the integrated electrochromic layers operate well within their thermal stability range without requiring active cooling. Since the ECD itself is too thin to effectively absorb electromagnetic waves, an FR4 substrate is attached beneath the device to introduce dielectric loss and enhance microwave absorption. Camouflage in the visible and NIR regions is based on reflectance control, therefore, the opaque FR4 substrate does not affect performance in these spectral domains.

### 2.2 Camouflage in Radar

To model the electromagnetic behavior of an optically thin absorber, a normally incident plane wave is considered. In the absence of a bulk volumetric response, the electromagnetic interaction is governed entirely by the induced electric and magnetic surface currents, $J_{e}$ and $J_{m}$, respectively. For a surface to achieve perfect absorption, these induced surface currents must cancel both the reflected and transmitted fields. This condition requires that the electric and magnetic surface currents satisfy the following relationship [38]:

(1) $$\eta_{0}J_{e}=\hat{z}\times J_{m},$$

where $\eta_{0}$ is the intrinsic impedance of free space and $\hat{z}$ denotes the unit vector normal to the surface. This relationship indicates that the absorber behaves as a Huygens' surface, in which the electric and magnetic responses are engineered to destructively interfere with the scattered waves in both the forward and backward directions.

Assuming a plane‐wave incidence, the electric and magnetic surface currents can be expressed as:

(2) $$J_{e}=\frac{1}{\eta_{0}}E_{\text{inc}},J_{m}=\eta_{0}H_{\text{inc}}.$$

To quantitatively characterize the absorber, its effective surface impedance $Z_{\text{surf}}$ as the ratio of the total tangential electric field ($E_{∥}$) to the electric surface current ($J_{e}$) [39]:

(3) $$Z_{\text{surf}}=\frac{E_{∥}}{J_{e}}=-j\eta_{0}\;\sin\left(k_{0}d\right)e^{jk_{0}d},$$

where d is the thickness of the absorbing layer and $k_{0}$ is the free‐space wave number.

The absorptivity *A* defined as the fraction of incident power that is neither reflected nor transmitted, can be expressed as:

(4) A(ω)=1−Γ(ω)−Τ(ω),

where Γ and Τ represent the reflection and transmission coefficients, respectively. Substituting the impedance‐matching expressions gives [40]:

(5) $$\Gamma \left(\omega \right)=\frac{Z_{\text{surf}}\left(\omega \right)-Z_{0}}{Z_{\text{surf}}\left(\omega \right)+Z_{0}},T\left(\omega \right)=\frac{2Z_{\text{surf}}\left(\omega \right)}{Z_{\text{surf}}\left(\omega \right)+Z_{0}}.$$

Perfect absorption occurs when $Z_{\text{surf}}\left(\omega \right)=Z_{0}$, yielding Γ(ω)=0 and T(ω)=0. Under this condition, all incident electromagnetic energy is absorbed by the surface. Here, $Z_{0}$ denotes the intrinsic impedance of free space (approximately 377 Ω). Based on this theoretical framework, the absorber was designed to achieve efficient absorption of incident electromagnetic waves, as illustrated in Figure 2a.

**FIGURE 2:**
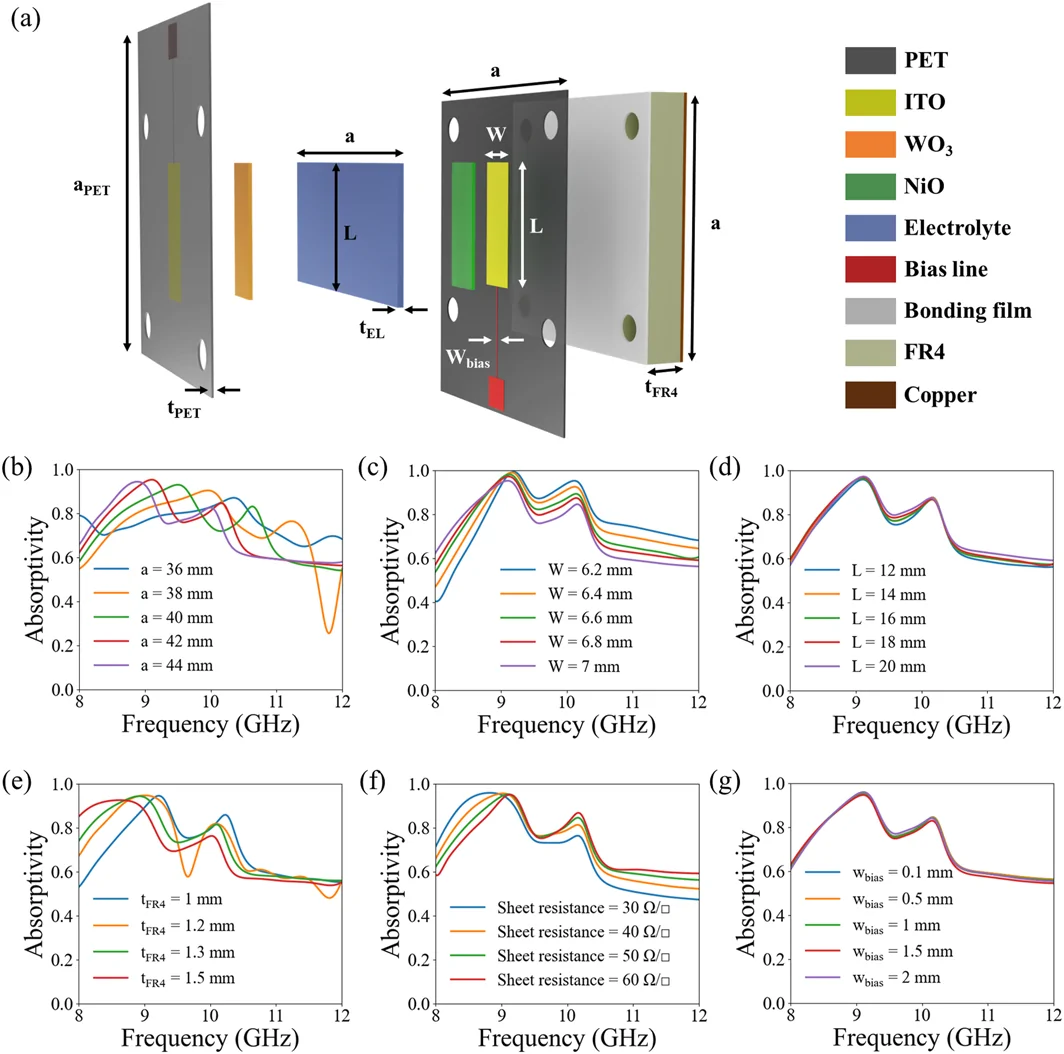
(a) Schematic of the proposed multispectral stealth device. Simulated absorptivity results showing the effects of variations in (b) substrate size, (c) ITO width (*W*), (d) ITO length (*L*), (e) FR4 substrate thickness (*t*_FR4_), (f) ITO sheet resistance, and (g) bias‐line width (*W*_bias_).

To ensure strict consistency between the simulations and experimental measurements, the numerical model was constructed based on the actual WR‐90 rectangular waveguide geometry rather than an infinite periodic structure with periodic boundary conditions. The excitation and boundary settings correspond to the dominant TE10 mode used in the measurements. For accurate verification, the same waveguide environment was replicated using the ANSYS High Frequency Structure Simulator (HFSS), as shown in Supporting Information S1: Figures S1 and S2.

As illustrated in Figure 2b, varying the substrate parameter (*a*) from 36 mm (smaller than the X‐band waveguide flange size) to 44 mm (larger than the flange) resulted in a downward shift of the resonant frequency. This shift occurs because the waveguide must satisfy boundary conditions defined by the flange dimensions; deviations from these conditions alter the electromagnetic response. Accordingly, *a* was optimized at 42 mm—matching the flange size—to preserve consistent boundary conditions and ensure reliable device performance. The geometric design of the patterned ITO was strategically optimized to satisfy both electromagnetic and optical requirements. Specifically, the unit cell was designed as a rectangular patch maximized to closely fit the waveguide aperture dimensions. This maximized structural design serves a dual purpose. First, from a microwave and fabrication perspective, minimizing the gap regions significantly reduces manufacturing tolerances and mitigates unwanted edge‐diffraction effects during waveguide characterization, ensuring high fidelity to the simulated impedance matching conditions. Second, from an optical perspective, maximizing the continuous area of the ITO pattern directly enlarges the active functional area of the underlying electrochromic layers. Because the Vis‐NIR modulation strictly occurs within the regions covered by the conductive ITO electrodes, increasing this geometric fill factor fundamentally maximizes the overall optical modulation depth, thereby optimizing the device’s multispectral camouflage performance.

As shown in Figure 2c, varying the ITO width (*W*) from 6.2 to 7 mm within the WR90 waveguide aperture (22.8 × 10.1 mm) caused a noticeable shift in resonant frequency. In contrast, as shown in Figure 2d, variations in ITO length (*L*) produced no significant effect. This is attributed to the dominance of the TE10 mode within the X‐band waveguide, in which the absorption performance is more sensitive to changes in *W* (aligned with the electric field direction) than to *L* (oriented perpendicular to it). Based on these findings, *W* was optimized to 6.8 mm to ensure efficient absorption within the X‐band. Additionally, *L* was set to 20 mm to enhance optical visibility, as a larger ITO area improves optical response while remaining within the WR90 dimensional constraints.

To ensure sufficient loss for electromagnetic wave absorption, a dielectric substrate with adequate thickness is required. In the ECD structure, the dielectric layers consist of two PET films, an electrolyte, and the WO_3_ and NiO_x_ layers, with respective thicknesses of *t*_PET_ = 175 μm, *t*_EL_ = 50 μm, and 300 nm each for the WO_3_ and NiO_x_ layers. In the proposed structure, the PET layer serves as an optically transparent and mechanically stable substrate for the electrochromic device, rather than as an electromagnetic tuning layer [41]. Consequently, its thickness was fixed according to commercially available ITO–PET substrates and was not treated as a design variable in the microwave optimization. This results in a total thickness of approximately 400.6 μm, which is insufficient to provide the dielectric loss required for effective absorption. To overcome this limitation, an FR4 substrate was attached beneath the ECD using a 35 μm‐thick bonding film, thereby increasing the total dielectric thickness and enhancing absorption performance. As shown in Figure 2e, varying the FR4 substrate thickness (*t*_FR4_) from 1 to 1.5 mm resulted in a shift in the resonant frequency. This shift occurs because the effective permittivity increases with substrate thickness, since the resonant frequency is inversely proportional to the square root of the effective permittivity, a thicker substrate leads to a lower resonant frequency. The FR4 layer acts as the key dielectric spacer for phase control and impedance matching in the X‐band, and its thickness was therefore systematically optimized to achieve maximum absorption.

In the ECD structure, the dielectric properties—specifically, the dielectric constant and dielectric loss—of both the PET layer, which is the thickest component, and the FR4 substrate must be considered in the design, as illustrated in Supporting Information S1: Figure S3. Based on these factors, the optimal *t*_FR4_ was determined to be 1.2 mm. As shown in Figure 2f, variations in the sheet resistance of the ITO layer, from 30 to 60 Ω/sq, affected the absorptivity. This behavior arises from changes in resistive losses, which are dependent on material conductivity and influence energy dissipation. However, the resonance frequency remained largely unaffected. To ensure fast electrochromic switching, ITO layers with relatively low sheet resistance were utilized. As demonstrated by the broadband transmittance measurements in Supporting Information S1: Figure S10, the ITO substrate maintains high transparency in the Vis–NIR range, whereas its transmittance is drastically reduced at longer infrared wavelengths. Therefore, the lack of MWIR and LWIR tunability represents a deliberate optimization trade‐off between electrical conductivity and broadband infrared transparency, rather than a structural deficiency.

To apply a DC voltage to the ITO and drive the ECD, a bias line is required that delivers the DC voltage while minimizing RF leakage. The bias line was therefore designed according to microstrip theory, as described in Supporting Information S1: Equations S2 and S3. As shown in Figure 2g, varying the bias‐line width (*W*_bias_) from 0.1 to 2 mm had negligible impact on absorption performance. This is because, within the X‐band waveguide, the dominant TE10 mode has its electric field propagates along the *y*‐axis; thus, the bias line was positioned perpendicular to this direction to minimize electromagnetic interference. The simulation results confirm that the patterned ITO enables effective radar camouflage while maintaining absorption efficiency and simultaneously allows DC bias application for visible and NIR electrochromic modulation.

As shown in Figure 3a, the electric field is primarily concentrated within the patterned ITO region, with minimal distribution near the bias line. This confirms that the ECD can operate without significantly affecting the absorber's electromagnetic performance. Figure 3b illustrates the electric‐field distribution across the ECD and FR4 substrate, where strong field confinement within the patterned ITO region confirms its dominant role in the resonance behavior. The surface loss density, presented in Figure 3c, further supports this finding, as most of the power dissipation per unit area occurs in the ITO region, verifying efficient electromagnetic absorption.

**FIGURE 3:**
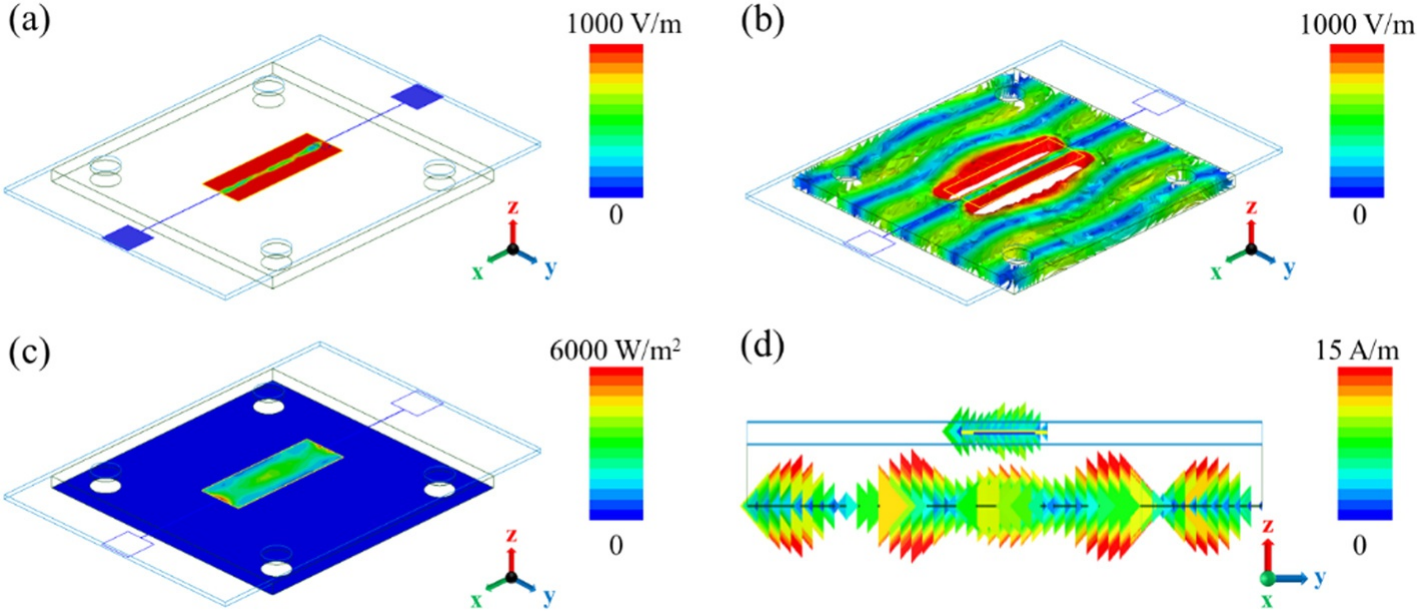
(a) Electric‐field distribution on the ITO layer and bias line. (b) Electric‐field distribution within the substrate. (c) Surface loss density. (d) Surface current density.

Figure 3d shows the surface current density on both the ITO and ground planes. The opposing directions of the surface currents indicate the formation of an inductive loop, confirming that the structure functions as a metamaterial‐based absorber with LC‐resonant circuit characteristics. Analysis of the electric‐field distribution, surface loss density, and surface current density at the resonance frequency of 9.1 GHz demonstrates that the proposed multispectral stealth device is effectively optimized for radar camouflage.

### 2.3 Camouflage in Visible Light and Near‐Infrared

As shown in Figure 4a, the ECD operates through redox reactions within electrochromic materials induced by an applied external voltage. This voltage drives the migration of lithium ions (Li^+^) from the electrolyte into the electrochromic layer. The resulting ion insertion triggers reduction (coloration) or oxidation (bleaching) reactions, thereby modifying the material's electronic structure and producing a measurable change in color or optical transparency.

**FIGURE 4:**
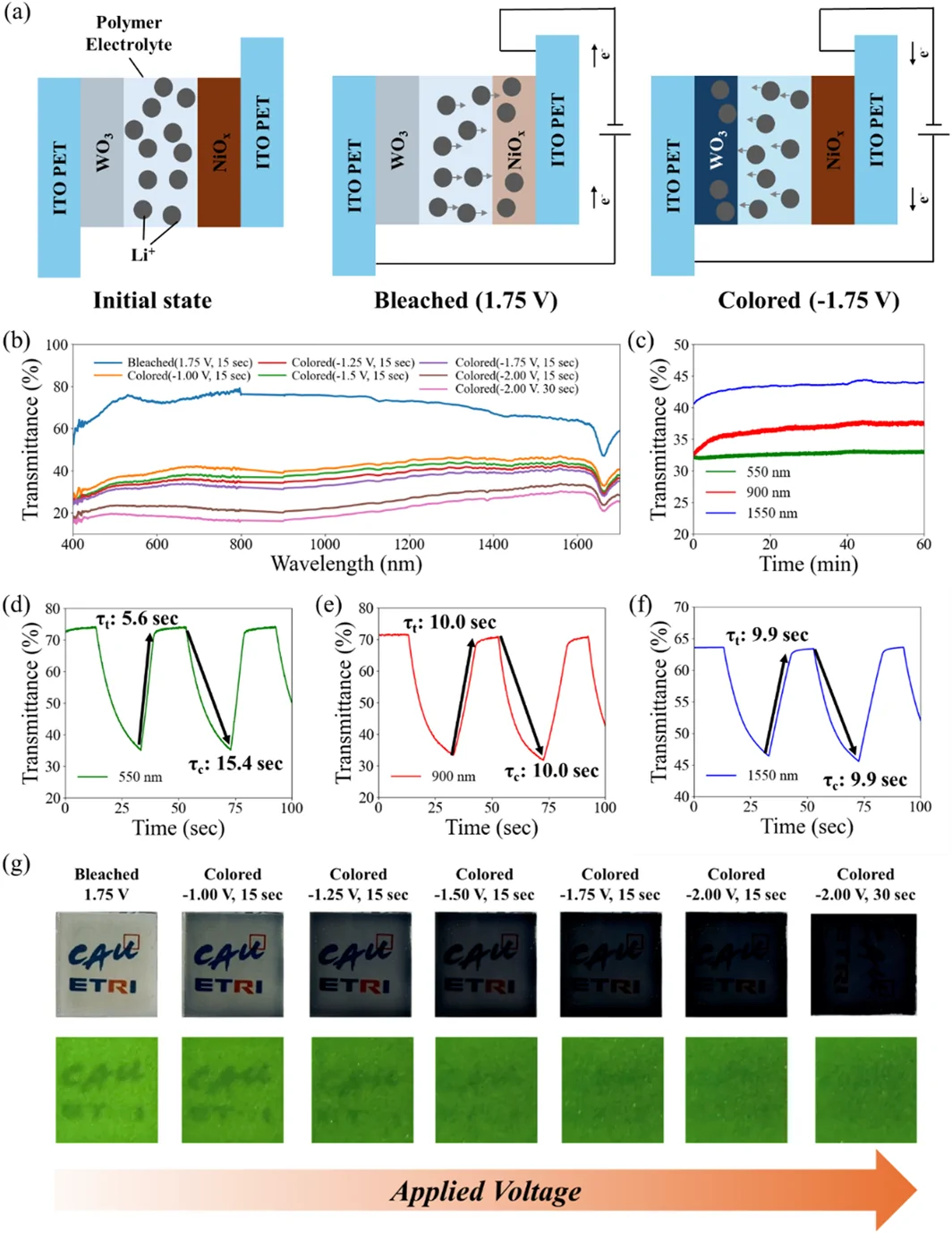
(a) Schematic illustration of the ECD operation in the bleached and colored states. (b) Voltage‐dependent transmittance. (c) Bistability measured at 550, 900, and 1550 nm under open‐circuit conditions following coloration. Switching speeds of the fabricated ECD measured at (d) 500, (e) 900, and (f) 1550 nm for coloration and bleaching. (g) Optical and IR images showing visible and thermal camouflage under various bias conditions.

To fabricate the ECD, two half‐cells were prepared separately by depositing WO_3_ and NiO_x_ onto ITO substrates. Surface SEM images (Supporting Information S1: Figure S9a,b) of the as‐deposited WO_3_ and NiO_x_ films reveal uniform and dense morphologies without visible defects such as cracks or pinholes, confirming high film quality. XRD patterns (Supporting Information S1: Figure S9c) of the WO_3_/glass and NiO_x_/glass samples exhibit no distinct crystalline peaks beyond the broad amorphous halo of the glass substrate, indicating that the deposited films are predominantly amorphous. For electrochromic materials, such amorphous structures are advantageous, as their disordered network provides more accessible pathways for Li^+^ ion insertion/extraction compared to crystalline phases, thereby enhancing coloration efficiency and cycling stability [42, 43, 44]. The electrochemical characteristics of each electrode were evaluated using cyclic voltammetry (CV), confirming their tunable optical transmittance (Supporting Information S1: Figure S4). These half‐cells were then assembled into a complete ECD using the PVB‐based gel electrolyte (Supporting Information S1: Figure S5). In addition, the PVB‐based gel electrolyte employed in this work offers a clear pathway toward scalable production. Although lab‐scale devices are prepared via casting, the same electrolyte formulation can be readily adapted to continuous roll‐to‐roll (R2R) processes, such as slot‐die coating, which are commonly used for large‐area electrochromic and optoelectronic devices. These scalable fabrication routes provide a realistic pathway toward large‐area implementations required for practical multispectral stealth applications. Upon voltage application, ion migration occurs according to the electrochemical reactions described in Supporting Information S1: Equations S4 and S5. The optical switching behavior of the assembled device is presented in Figure 4b. At 550 nm, transmittance varies from 74.5% in the bleached state (1.75 V for 15 s) to 18.9% in the colored state (−2.0 V for 30 s), corresponding to a modulation depth of 55.6% in the visible spectrum. Similarly, at 900 nm, the modulation depth is 59.8%, confirming dynamic optical control in the NIR range. These results demonstrate that the ECD provides precise, voltage‐dependent optical tuning across both spectral domains, highlighting its potential for adaptive camouflage applications. The bistability of the device is shown in Figure 4c. After coloration, the ECD retained its optical state under open‐circuit conditions for 30 min, with transmittance variations below 0.4%, 4.4%, and 2.6% at 550, 900, and 1550 nm, respectively. This stability indicates that the device can maintain its optical state without continuous power input.

Combined with its low operating voltage, this bistability makes the ECD well suited to low‐power camouflage and surveillance platforms operating in energy‐constrained environments.

To assess the cycling stability of the device, repeated electrochemical switching was performed under a fixed voltage protocol of −1.75 V for 15 s (coloration) and +1.75 V for 15 s (bleaching). As shown in Supporting Information S1: Figure S6, the average optical modulation across the visible and IR regions was 39.1% after the first cycle and 38.4% after 1000 cycles. The negligible degradation (< 2%) in optical contrast demonstrates excellent electrochemical reversibility and optical durability during prolonged operation.

When a voltage is applied, the device colors rapidly, while reversing the polarity induces prompt bleaching. As shown in Figure 4d, at 550 nm the bleaching time, defined as the time to reach 90% of maximum transmittance from the colored state, is 5.6 s, whereas the coloration time, defined as the time to achieve 90% of maximum coloration from the bleached state, is 15.4 s. At 900 nm (Figure 4e), both bleaching and coloration occur within 10 s, and at 1550 nm (Figure 4f), both transitions take approximately 9.9 s. As demonstrated in Figure 4g, effective camouflage in both the visible and NIR spectral ranges is achieved within 30 s of voltage application. To establish a quantitative relationship between electrochemical activity and optical response, the ECD was further characterized using chronoamperometry (CA) and chronocoulometry (CC) measurements (Supporting Information S1: Figure S7). These measurements confirm stable switching behavior and provide the basis for calculating the coloration efficiency (CE), defined as the change in optical density (ΔOD) per unit of inserted charge. A higher CE value indicates greater optical modulation for a smaller charge. As shown in Supporting Information S1: Figure S8, ΔOD as a function of inserted charge yielded CE values of 91.8, 95.1, and 50.0 cm^2^ C^−1^ at 550, 900, and 1550 nm, respectively. These results confirm that the ECD provides efficient, voltage‐controlled optical modulation across both the visible and IR regions, validating its effectiveness for multispectral camouflage.

### 2.4 Experiment Results of Multispectral Stealth

Figure 5a shows the measured microwave absorptance of the device in the unbiased state. The measurements were conducted using a waveguide setup, in which the reflection and transmission coefficients were first obtained and subsequently substituted into Equation (4) to calculate absorptance. The results reveal that the device achieves over 90% absorptance across the 8.7–9.3 GHz frequency range, demonstrating excellent agreement with simulation data.

**FIGURE 5:**
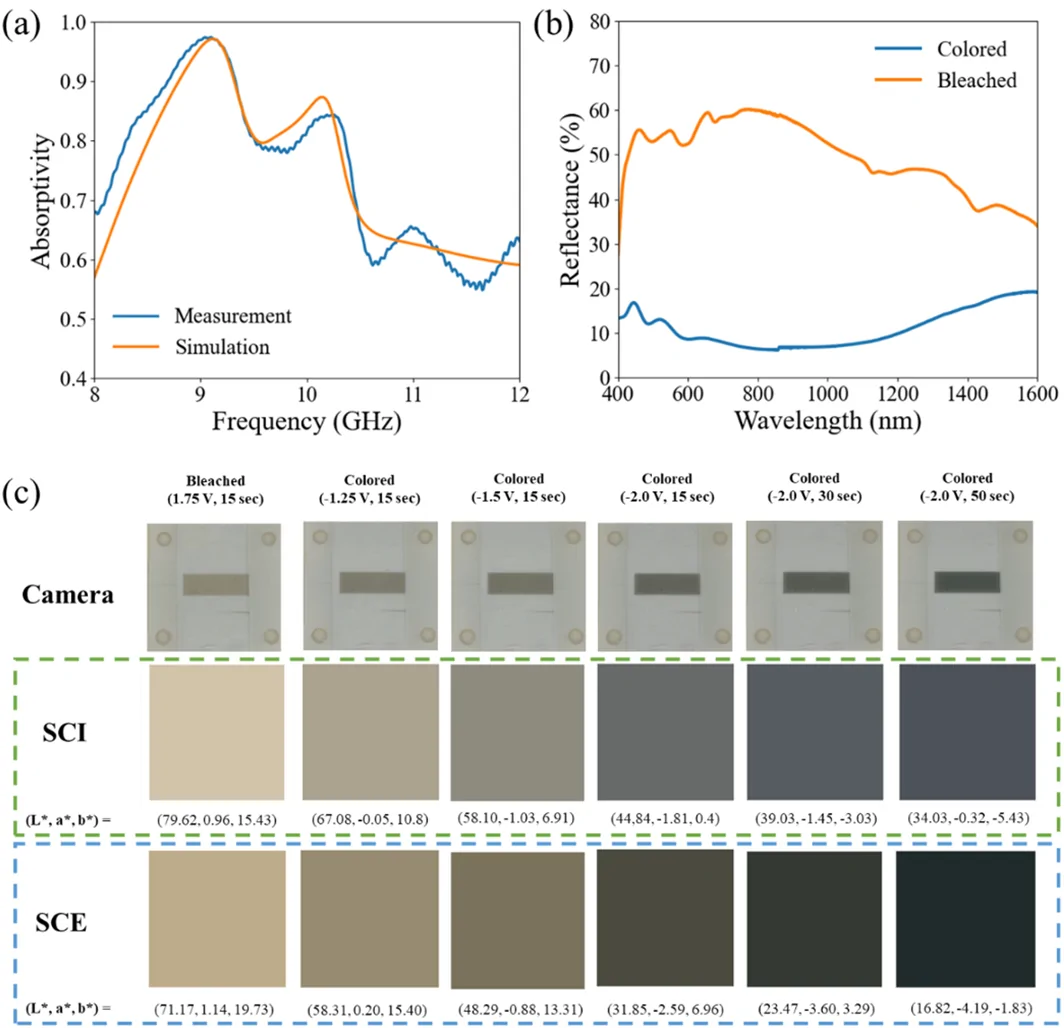
(a) Comparison between simulated and measured absorptivity in the microwave regime. (b) NIR reflectance measurement. (c) SCE (actual color perceived by the observer, excluding specular reflection) and SCI (including specular reflection, according to colorimetric standards) values measured using a spectrophotometer.

Figure 5b presents the wavelength‐dependent reflectance spectra of the multispectral stealth device under applied voltage in both the colored and bleached states. Measurements were performed in diffuse reflectance mode using a UV–VIS/NIR spectrophotometer over the 0.4–1.7 μm range. Under coloration (−2 V, 30 s) and bleaching (1.75 V, 15 s) conditions, a clear spectral contrast was observed between the two states, yielding an average reflectance modulation of 37.4% across the entire VIS–NIR region. The reflectance modulation in the NIR range was further verified through IR detection tests using a commercial 900 nm sensor module (Supporting Information S1: Figure S12), which confirmed that the device's switching behavior effectively controlled the detection response of the sensor.

Figure 5c displays both photographic images and colorimetric coordinates (L*, a*, b*) of the device measured under various applied voltages in SCI (specular component included) and SCE (specular component excluded) modes. As the voltage magnitude and duration increase, the device progressively transitions from the bleached to the colored state. During this process, the a* and b* values remain nearly constant, whereas the L* value decreases substantially. For instance, in SCI mode, the L* value decreases from 79.62 in the bleached state (1.75 V, 15 s) to 34.03 in the fully colored state (−2.0 V, 50 s). In SCE mode, it decreases from 71.17 to 16.82 under the same conditions. These results demonstrate that the device selectively modulates brightness (L*) with minimal chromaticity change, effectively functioning as an optical shutter for visibility control and light attenuation. Such characteristics enable precise control of brightness contrast relative to the background under varying illumination conditions, offering significant advantages for visible‐spectrum camouflage applications.

Taken together, these results confirm that the device exhibits multifunctional tunability across the microwave, NIR, and visible regimes, enabling efficient electromagnetic absorption, NIR reflectance modulation, and high‐contrast optical switching.

Table 1 provides a comparative summary of recent multispectral stealth studies encompassing the visible, infrared (IR), and microwave domains. The comparison highlights key performance metrics, including visible camouflage capability, IR emissivity or reflectance across relevant spectral bands, microwave stealth performance, tuning mechanism (active or passive), response time, number of spectral bands covered, and total structural thickness.

**TABLE 1:** Comparison between the results obtained in this study and those obtained in previous multispectral stealth device based studies works.

Ref.	Visible camouflage	IR camouflage		Microwave camouflage	Tuning method	Number of spectrum	Response time	Total thickness
		Wavelength (µm)	Emissivity or reflectance					
[45]	No	8–14	0.13 (emissivity)	Yes	Active	2	30 ms	4.5 mm
[46]	No	8–14	0.14 (emissivity)	Yes	Passive	2	50 s	2.4 mm
[47]	No	3–5, 8–14	0.14, 0.22 (emissivity)	Yes	Passive	2	N/A	9 mm
[48]	Visible transparent	3–5, 8–14	0.36, 0.35 (emissivity)	Yes	Passive	3	N/A	7.35 mm
[49]	Color‐based camouflage	3–5, 8–14	0.11, 0.12 (emissivity)	Yes	Passive	3	N/A	2.35 mm
This work	Color‐based camouflage	0.75–1.4	0.094 (reflectance)	Yes	Active	3	5.6 s	1.67 mm

Previous studies exhibit diverse design strategies and performance trade‐offs. Several approaches do not incorporate visible camouflage, whereas others achieve either visible transparency or color‐based concealment under visual observation. In the IR domain, most reported works focus on the mid‐wave (3–5 μm) and long‐wave (8–14 μm) infrared bands, achieving emissivity values typically ranging from 0.11 to 0.36. Although low emissivity effectively suppresses thermal radiation, many of these systems rely on passive mechanisms or multilayer stacking architectures, which often results in increased structural thickness and limited dynamic adaptability. Furthermore, passive designs inherently lack real‐time tunability once fabricated.

In contrast, the technology presented in this work adopts an active electrochromic tuning strategy, enabling reversible and voltage‐controlled modulation in the visible and near‐infrared (NIR) regions, with a minimum reflectance of 0.094. Although electrochromic modulation generally exhibits response times on the order of seconds—slower than purely electronic switching—it offers a favorable balance between energy efficiency, stability, and controllability. Importantly, the integration of the electrochromic device does not compromise the excellent microwave absorption performance, which exceeds 90% in the X‐band.

From a system‐level perspective, the proposed monolithic architecture achieves triple‐band camouflage across the visible, IR, and microwave spectra within a compact total thickness of 1.67 mm, significantly reducing integration complexity compared to multilayer stacking approaches. Although further optimization is required to enhance response speed and large‐area scalability, the demonstrated balance among active tunability, multispectral coverage, and structural compactness highlights the distinct positioning of this work among current multispectral stealth platforms.

To ensure the practical deployment of the stealth device in complex real‐world environments, such as on mobile unmanned aerial vehicles (UAVs), both environmental durability and electromagnetic angular stability are critical requirements. Environmentally, the robustness of the device is primarily governed by the PVB‐based gel electrolyte, which provides strong adhesion, moisture resistance, and excellent thermal stability [50, 51, 52, 53]. As supported by literature, this ensures stable electrochromic operation across a wide temperature range (−20°C–80°C) and under high humidity conditions [54]. The device exhibited an optical modulation amplitude of 42.80% in the flat state. Under a bending radius of 30 mm, 91% of the modulation was retained, and even at a 20 mm radius, more than 80% of the modulation was preserved (Supporting Information S1: Figure S11). Performance degradation was observed at a 10 mm radius due to the intrinsic brittleness of the ITO electrode; however, no delamination or structural failure occurred, indicating that the PVB‐based electrolyte effectively maintains interfacial integrity under mechanical stress. Electromagnetically, although the present characterization was conducted in a waveguide configuration to rigorously validate the intrinsic absorption mechanism at normal incidence, maintaining broadband absorption under oblique free‐space illumination is equally essential [55, 56]. Therefore, future work will focus on large‐area fabrication and systematic free‐space angular measurements to fully evaluate and optimize the comprehensive stability of the proposed structure for real‐world mobile platforms.

## 3 Conclusions

Multispectral stealth spanning the visible, NIR, and radar spectra was successfully demonstrated under controlled waveguide conditions by integrating a voltage‐responsive electrochromic device (ECD) with a metamaterial absorber patterned using ITO on a shared substrate. Upon voltage application, the ECD achieved visible transmittance modulation from 74.5% to 18.9%, enabling dynamic visible camouflage, whereas simultaneously modulating NIR reflectance from 50.3% to 9.4%. Concurrently, the ITO‐patterned metamaterial structure provided radar camouflage by absorbing more than 90% of incident electromagnetic waves in the X‐band frequency range.

The waveguide‐based characterization, corresponding to normal‐incidence excitation under well‐defined boundary conditions, rigorously validates the intrinsic absorption mechanism and impedance matching behavior of the proposed architecture. Although the present radar evaluation was performed under controlled waveguide conditions for proof‐of‐concept verification, further investigations under free‐space illumination, oblique incidence, and realistic platform configurations will be necessary to fully assess practical deployment performance.

This all‐in‐one architecture successfully integrates distinct optical and electromagnetic mechanisms within a monolithic platform, demonstrating the feasibility of an integrated approach to multispectral stealth. With further optimization toward angular robustness, mechanical durability, environmental stability, and scalable fabrication, the proposed design offers significant potential for next‐generation adaptive defense and security applications.

## 4 Materials and Methods

### 4.1 Electromagnetic Simulation

Electromagnetic simulations were performed using the ANSYS HFSS. A straight WR90 waveguide (22.8 × 10.1 mm) was modeled, with two opposing waveguides positioned to hold the sample between them. Wave ports were assigned to excite the dominant TE1_0_ mode, whereas the outer boundaries were defined using a radiation box. To improve simulation efficiency, the patterned ITO mesh was represented as a surface resistance, and the multilayer ECD stack was approximated as a single, characterized effective medium.

### 4.2 ECD Fabrication

Prior to ECD fabrication, patterned ITO‐coated polyethylene terephthalate (PET) substrates (sheet resistance: 50 Ω sq^−1^) were sequentially cleaned in ultrasonic baths containing acetone and isopropanol. The ITO pattern proposed in this study was fabricated using a laser patterning process on an ITO PET substrate. Using a precisely controlled pulsed laser beam, only the ITO thin film layer was selectively removed according to the designed drawing, physically separating the conductive active region from the insulating pattern region. Thin films of WO_3_ and NiO_x_ were subsequently deposited onto the substrates via DC sputtering (SPARKLE‐2CM multi‐sputtering system, Unitex). A polyvinyl butyral (PVB)‐based polymer electrolyte was applied to the WO_3_‐coated electrode using the doctor‐blade method and dried in an oven at 80°C for 90 s to achieve a uniform thickness of approximately 40 μm. The NiO_x_‐coated counter electrode was then laminated onto the dried electrolyte under ambient conditions. The two electrodes were bonded by the intrinsic adhesiveness of the partially tacky polymer electrolyte, forming a self‐sealing electrochromic cell.

### 4.3 Absorption Measurement

Microwave absorption performance was characterized using two straight X‐band waveguides (SMF90S‐03) conforming to WR90 standard dimensions and supporting the fundamental TE10 mode. A Keysight N9951A FieldFox handheld vector network analyzer (frequency range: 300 kHz–44 GHz) was connected to the waveguides to measure the reflection (S11) and transmission (S21) coefficients of the electromagnetic waves transmitted through the sample. The absorptance was subsequently calculated from the measured S‐parameters according to the standard power‐balance relation.

### 4.4 Optical Measurement

The optical characteristics of the ECD and the EPD were measured using an optical measurement system comprising a UV–visible spectrometer (USB2000+UV–VIS, Ocean Optics), a fiber‐optic light source (DH‐2000‐BAL, Ocean Optics), and an NIR spectrometer (NIRQuest512, Ocean Optics). Reflectance measurements were carried out in diffuse reflectance mode using an integrating sphere attachment. A Spectralon white reflectance standard (SRS‐99‐010, Labsphere) was employed as a calibration reference to ensure accurate baseline correction. Electrochemical performance was evaluated using an electrochemical workstation (CH1030, CH Instruments). Voltage signals were supplied through an isolated control board equipped with an operational amplifier and a DC power supply (OPE‐303Q, Protek).

## Author Contributions

**Jinwoo Ho:** conceptualization, methodology, data curation, formal analysis, software, investigation, visualization, validation, resources, writing – review and editing, writing – original draft. **Juhyun Park:** conceptualization, methodology, data curation, formal analysis, software, validation, investigation, visualization, resources, writing – original draft, writing – review and editing. **Jiyoung Oh:** investigation, methodology. **Chil Seong Ah:** methodology, resources. **Jae Woo Seo:** investigation, validation. **HeaSook Park:** conceptualization, project administration, funding acquisition. **Tae‐Youb Kim:** funding acquisition, project administration, supervision. **Sungjoon Lim:** conceptualization, funding acquisition, writing – review and editing, project administration, supervision.

## Funding

This research was supported by the Challengeable Future Defense Technology Research and Development Program through the Agency For Defense Development (ADD) funded by the Defense Acquisition Program Administration (DAPA) in 2026 (Grant 915144201).

## Conflicts of Interest

The authors declare no conflicts of interest.

## Supporting information


Supporting Information S1

**Video S1:** Simultaneous visible and near‐infrared time‐lapse imaging of the ECD under varying applied voltages.

## Data Availability

Data sharing is not applicable to this article as no datasets were generated or analyzed during the current study.
