# Supplementary material for: All‐in‐One Multispectral Stealth Device for X‐Band Radar, Visible, and Near‐Infrared Spectra
Source: Nanophotonics. 2026 Apr 22;15(8):e70102. doi: 10.1002/nap2.70102 (PMC13642061; doi:10.1002/nap2.70102)
Supplement: Supplementary file 1 [file NAP2-15-e70102-s002.docx]

Supporting Information

All-in-One Multispectral Stealth Device for X-band Radar, Visible, and Near-Infrared Spectra

Jinwoo Ho^a^, Juhyun Park^b,c^, Juhyun Park^b,c^, Jiyoung Oh^c^, Chil Seong Ah^c^, Jae Woo Seo^c,d^, HeaSook Park^c^, Tae-Youb Kim^c*^, and Sungjoon Lim^a*^

^a^ Department of Intelligent Semiconductor Engineering, Chung-Ang University, Seoul 06974, Republic of Korea

^b^ Display and Nano System Laboratory, School of Electrical Engineering, Korea University, Seoul 02841, Republic of Korea

^c^ Reality Devices Research Division, Electronics and Telecommunications Research Institute, Daejeon 34129, Republic of Korea

^d^ Division of Materials Science and Engineering, Hanyang University, Seoul 04763, Republic of Korea

*E-mail: youby@etri.re.kr, [sungjoon@cau.ac.kr](mailto:sungjoon@cau.ac.kr)

**Note S1 Preparation of the X-band waveguide.**

Waveguides are enclosed by metallic walls, which effectively shield them from external noise and minimize signal attenuation within the structure, thereby maintaining strong signal integrity. Owing to their ability to support only specific propagation modes, waveguides also suppress undesired modes and mitigate external interference. The X-band waveguide used in this study conforms to the WR90 standard. The cut-off frequency for a rectangular waveguide with dimensions $a$ and $b$ is given by:

$$\begin{aligned} f_{c\left( mn \right)}=\frac{c}{2}\sqrt{\left( \frac{m}{a} \right)^{2}+\left( \frac{n}{b} \right)^{2}}\#\left( S1 \right) \end{aligned}$$

where $m$ and $n$ denote the mode indices, and $c$ is the speed of light in vacuum. For the dominant TE_10_ mode ($m$ = 1, $n$ = 0), the expression simplifies to $f_{c}=c/2a$, which yields a cut-off frequency of 6.56 GHz for the WR90 waveguide. Based on these characteristics, two straight X-band waveguides were modeled in ANSYS HFSS, as illustrated in Figure S1a and S1b.

**Figure S1.** (a) Top waveguide, (b) bottom waveguide, (c) dimensions of the WR-90 waveguide, (d) dimensions of the straight waveguide, (e) simulation environment.

The dimensions were set to match those of the WR90 waveguides used in the measurements, as shown in Figure S1c and S1d. As illustrated in Figure S1e, wave ports were applied to both the upper and lower ends of the combined waveguide structure for simulation.

Figure S2a and S2b compare the simulated and measured results for the waveguides without any sample inserted. The close agreement between the simulated and experimental S_11_ and S_21_ parameters confirms that the simulated environment accurately replicates the actual measurement setup. As shown in Figure S2c, simulation results further verify that the TE10 mode propagates through the waveguides as intended.

**Figure S2.** (a) S11 and (b) S21. (c) E-field distribution in X-band waveguides.

**Note S2 Dielectric properties of the ECD and FR4 substrate.**

**Figure S3.** (a) Absorptivity as a function of the relative permittivity of the ECD. (b) Absorptivity as a function of the relative permittivity of the FR4 substrate.
(c) Absorptivity as a function of the loss tangent of the ECD. (d) Absorptivity as a function of the loss tangent of the FR4 substrate.

Figure S3 presents the simulated absorptance spectra of the ECD-based absorber under variations in the dielectric properties of the ECD layer and the FR4 substrate. As shown in Figure S3a and S3b, increasing the dielectric constant (ε) of either the ECD or FR4 layers results in a red-shift of the resonance frequency due to the increased effective electrical length. Figure S3c and S3d illustrate the impact of dielectric loss (tan δ) on absorptance. As the loss increases, the resonance peaks broaden, and the overall absorption slightly increases in non-resonant regions, indicating a reduced Q-factor. The effect of loss variation in the ECD layer is more pronounced than that in the FR4 substrate. These results provide practical guidelines for tuning the resonance frequency and absorption strength through material parameter optimization.

**Note S3 Bias line design method based on microwave engineering theory**

In microwave engineering, DC bias lines are integrated into high-frequency circuits to deliver a constant voltage or current to active components without interfering with the RF signal path. To suppress RF leakage through the bias line, high-impedance structures—such as quarter-wavelength open stubs or radial stubs—are commonly used as RF chokes. These structures present high impedance at the operating frequency, thereby effectively isolating the DC path from the RF signal. Additionally, decoupling capacitors are strategically placed to provide low-impedance paths for RF signals to ground, further minimizing distortion. Thus, the design of DC bias lines requires precise impedance matching and careful layout optimization to ensure minimal degradation of the circuit’s electromagnetic performance.

$$\begin{aligned} Z_{0}\propto\frac{1}{W}\#\left( S2 \right) \end{aligned}$$

$$\begin{aligned} Z_{\text{in}}=Z_{0}\cdot\frac{Z_{L}+jZ_{0}\tan\left( \beta l \right)}{Z_{0}+jZ_{L}\tan\left( \beta l \right)}\#\left( S3 \right) \end{aligned}$$

According to Equation S2, a narrower bias line results in higher characteristic impedance (Z₀), which helps suppress RF signal leakage into the DC path. From Eq. S3, when $l=\lambda/4$, the input impedance (Z_in_) approaches infinity, thereby effectively isolating the RF signal and preventing its entry into the DC bias line.

**Note S4 Preparation of the half-cell ECD**

The electrochromic performance of the proposed ECD was evaluated based on its spectral tunability across the VIS and IR regions, electrochemical responsiveness, and bistability. Figure S4a and S4b present the CV profiles of the WO_3_ and NiO_x_ half-cells, respectively, under various scan rates. WO_3_ exhibited characteristic cathodic behavior, with increasing reduction current at negative potentials, whereas NiO_x_ demonstrated anodic behavior, marked by increased oxidation current at positive potentials. The associated redox reactions are represented as follows:

$$\begin{aligned} \left. Liₓ\text{WO₃ (Blue)}\rightleftarrows xe^{-}+x{Li}^{+}+\text{WO₃} (Transparent \right)\#\left( S4 \right) \end{aligned}$$

$$\begin{aligned} \left. Li_{2-x}{NiO}_{x}\text{ (Brown) + }xe^{-}\rightleftarrows+x{Li}^{+}+Li_{2}{NiO}_{x} (Transparent \right)\#\left( S5 \right) \end{aligned}$$

The corresponding optical modulation of each electrode under voltage bias is shown in Figure S4c and S4d. Upon reduction, WO_3_ transitions from a transparent to a blue state, accompanied by a substantial decrease in transmittance across both the VIS and IR spectral regions. In contrast, NiO_x_ changes from transparent to brown upon oxidation, but its modulation in the IR range is relatively limited compared to that of WO_3_, indicating that WO_3_ plays a dominant role in IR spectral control. When integrated into a complete ECD, the combined coloration of blue (WO_3_) and brown (NiO_x_) yields an optically darkened (black-like) or highly transparent state, thereby enabling dynamic contrast control suitable for effective camouflage.

**Figure S4.** (a) Cyclic voltammetry and (b) transmittance in the colored and bleached states of the NiO_x_ half-cell. (c) Cyclic voltammetry (CV) and (d) transmittance in the colored and bleached states of the WO_3_ half-cell.

**Figure S5.** Wavelength-dependent transmittance of the PVB electrolyte.

**Note S5 Preparation of the full-cell ECD**

**Figure S6.** Comparison of transmittance between a single switching cycle and after 1000 repeated cycles of coloring (−1.75 V, 15 s) and bleaching (+1.75 V, 15 s).

To assess the long-term optical durability of the electrochromic device (ECD), the transmittance spectra were measured in both the colored and bleached states after the initial switching cycle and after 1,000 full coloration/bleaching cycles, as shown in Figure S6.

After 1,000 cycles, the bleached-state transmittance (black dashed line) shows minimal degradation compared to the initial measurement (black solid line), indicating excellent optical retention and material stability. After 1,000 cycles, the red dashed line indicates that the optical contrast in the colored state is also well-maintained, with only slight variation, confirming the robust electrochromic performance of the device.

These results verify the ECD’s strong cyclability and endurance, with negligible optical fatigue even after extended use.

**Figure S7.** (a) Chronoamperometry (CA) and (b) chronocoulometry (CC) measurements of the fabricated ECD.

To verify the electrochemical switching behavior of the fabricated electrochromic device (ECD), time-dependent current density and the corresponding charge density were measured during repeated voltage cycling.

Figure S7a presents the current density profile over time under a square-wave potential modulation. Alternating anodic and cathodic current peaks were observed, corresponding to the bleaching and coloration processes of the ECD, respectively.

Figure S7b displays the integrated charge density over time, obtained by time-integration of the current density curves. The periodic increase and decrease in charge density confirm the consistent ion insertion and extraction processes associated with electrochromic switching.

**Figure S8.** Coloration efficiency of the ECD at wavelengths of 550, 900, and 1550 nm.

To evaluate the performance of the electrochromic device (ECD), the coloration efficiency (CE) was investigated by analyzing the optical density change (ΔOD) as a function of charge density (mC/cm²) at three representative wavelengths: 550 nm, 900 nm, and 1550 nm. Coloration efficiency is defined as the optical contrast achieved per unit of injected or extracted charge, indicating how effectively the ECD modulates light with minimal energy consumption. A higher CE value implies more efficient electrochromic behavior.

The CE values were extracted by linear fitting of the ΔOD versus charge density data, following the relationship:

$$\begin{aligned} \triangle OD= \log\left( \frac{T_{b}}{T_{c}} \right)\#(S5) \end{aligned}$$

$$\begin{aligned} CE= \triangle\frac{OD}{Q_{d}}\#\left( S7 \right) \end{aligned}$$

**Figure S9.** Comparative analysis of SEM image of (a) WO3/Glass, (b) NiOx/Glass samples and (c) XRD graph.

**Figure S10.** Transmittance by ITO thickness in (a) Vis-IR, (b) MWIR, (c) LWIR

**Figure S11.** Bending test result

**Note S6 Infrared detection measurements conducted using a Raspberry Pi.**

**Figure S12.** Experimental setup for infrared object detection during electrochromic device (ECD) switching.

To experimentally demonstrate the near-infrared modulation capability of the fabricated electrochromic device (ECD), a test setup was constructed comprising the ECD, a commercial infrared object detection sensor (comprising both emitter and receiver operating at 900 nm), a Raspberry Pi for data processing, and a regulated power supply. The sensor module includes a visible green indicator LED that signals object detection status.

As illustrated in Figure S9, the presence of an object was inferred based on the sensor's ability to receive reflected IR light. In the bleached state, the ECD permits IR transmission and subsequent reflection from the object, allowing the sensor to detect its presence and activate the indicator light. Conversely, in the colored state, the ECD absorbs a significant portion of the incident IR light around 900 nm, preventing it from reaching the object and reflecting back. As a result, the sensor fails to detect any reflected signal, thus registering the object as absent.

**Video S1.** Simultaneous visible and near-infrared time-lapse imaging of the ECD under varying applied voltages.
